# Feasibility of implementing routine nutritional screening for older adults in Australian general practices: a mixed-methods study

**DOI:** 10.1186/s12875-014-0186-5

**Published:** 2014-11-25

**Authors:** Aliza Haslinda Hamirudin, Karen Charlton, Karen Walton, Andrew Bonney, Jan Potter, Marianna Milosavljevic, Adam Hodgkins, George Albert, Abhijeet Ghosh, Andrew Dalley

**Affiliations:** School of Medicine, Faculty of Science, Medicine and Health, University of Wollongong, Wollongong, NSW 2522 Australia; Illawarra and Southern Practice Research Network, Graduate School of Medicine, Building 28, University of Wollongong, Wollongong, NSW 2522 Australia; Illawarra Shoalhaven Local Health District, Wollongong Hospital, LMB 8808, South Coast Mail Centre, NSW 2521 Australia; Illawarra Shoalhaven Medicare Local, Suite 3, Level 1, 336 Keira Street, Wollongong, NSW 2500 Australia; Illawarra Health and Medical Research Institute, Wollongong, NSW Australia

**Keywords:** Malnutrition, Older adults, Nutrition screening, Training, Feasibility

## Abstract

**Background:**

Nutrition screening in older adults is not routinely performed in Australian primary care settings. Low awareness of the extent of malnutrition in this patient group, lack of training and time constraints are major barriers that practice staff face. This study aimed to demonstrate the feasibility of including a validated nutrition screening tool and accompanying nutrition resource kit for use with older patients attending general practice. Secondary aims were to assess nutrition-related knowledge of staff and to identify the extent of malnutrition in this patient group.

**Methods:**

Nine general practitioners, two general practice registrars and 11 practice nurses from three participating general practices in a rural, regional and metropolitan area within a local health district of New South Wales, Australia were recruited by convenience sampling.

Individual in-depth interviews, open-ended questionnaires and an 11-item knowledge questionnaire were completed three months following in-practice group workshops on the Mini Nutritional Assessment Short Form (MNA-SF). Staff were encouraged to complete the MNA-SF within the Medicare-funded 75+ Health Assessment within this time period. Staff interviews were digitally recorded, transcribed verbatim and analysed thematically using qualitative analysis software QSR NVivo 10.

**Results:**

Four key themes were determined regarding the feasibility of performing MNA –SF: ease of use; incorporation into existing practice; benefit to patients’ health; and patients’ perception of MNA-SF. Two key themes related to the nutrition resource kit: applicability and improvement. These findings were supported by open ended questionnaire responses. Knowledge scores of staff significantly improved from baseline (52% to 66%; P < 0.05). Of the 143 patients that had been screened, 4.2% (n = 6) were classified as malnourished, 26.6% (n = 38) ‘at risk’ of malnutrition and 69.2% (n = 99) as well-nourished.

**Conclusion:**

It is feasible to include the MNA-SF and a nutrition resource kit within routine general practice, but further refinement of patients’ electronic clinical records in general practice software would streamline this process.

## Background

The demographic group of adults aged 65 years and above have the highest frequency of consultations in General Practice in Australia, with the frequency of consultations increasing with age [1]. The BEACH (Bettering the Evaluation And Care of Health) study reported that between 2011 – 2012, patients aged 70 to 79 years had on average 12 General Practice interactions per year, which increased to 16 in those ≥85 years, as compared with about four per year in adults aged 25 to 49 years. It is estimated that adults aged ≥65 years account for 31% of all encounters in General Practice [2] and that these encounters have increased significantly in the last 10 years [3].

Malnutrition is an under-recognized threat to older adults’ health status which leads to numerous adverse health outcomes [4,5]. Older adults who are at nutritional risk have increased risk of illness [6], more frequent visits to their general practitioners [6], poorer quality of life [7] and higher mortality compared to their well-nourished counterparts [4]. The estimated prevalence of nutritional risk in community living older adults in Australia is approximately 45% [8,9].

In Australia, within general practice settings, the timely identification and management of malnutrition should be a focus of preventative health activities in older adults for improved quality of patient care [10]. The ‘Enhanced Primary Care’ (EPC) package that was introduced by the Commonwealth Department of Health and Aged Care in 1999 includes ‘Health assessments for adults aged 75 years and over’ (75+ HA) [11]. One of the aims of the EPC package was to improve older persons’ access to health services [12]. The 75 + HA includes evaluation of various medical and non-medical conditions including cognitive function, social status, activities of daily living (ADL), depression and mobility. However, a low uptake of this Medicare Benefits Scheme (MBS) funded item by age-eligible patients has been reported [13,14].

Clinical guidelines in Australia recommend screening for malnutrition in older adults across all health care settings, as well as in residential aged care facilities [10,15–18], but this does not routinely occur [19]. Within primary care, implementation of routine nutrition screening necessitates use of a validated instrument that is easy to use, practical in its application, and that provides results that can clearly be applied to clinical pathways and further referrals [20]. Many nutrition screening tools exist for use in older adults [21]. The 6-item Mini Nutrition Assessment Short Form (MNA-SF) [22] is an abbreviated and validated screen that has been adapted from a more comprehensive 18-item version [23]. The MNA-SF can be completed within five minutes [24] and is appropriate for community-dwelling older people [25,26].

In other countries, tackling malnutrition has been demonstrated to improve clinical outcomes and reduce health care use [27]. In the UK, malnourished adults have disease related malnutrition costs in excess of £13 billion per annum, based on malnutrition prevalence figures and associated costs of both health and social care [27]. It has been estimated that costs associated with malnutrition in European countries are more than twofold the costs related to obesity [28]. The National Institute for Health and Clinical Excellence has also demonstrated substantial cost savings associated with the implementation of a clinical guideline on malnutrition in adults [29].

Our earlier work (Phase 1 research) identified barriers faced by general practice staff that impact on the incorporation of nutrition screening in their interactions with older patients. These include time constraints, low prioritization of nutrition-related issues, cost of performing the activity and lack of knowledge and skills [30]. These findings identified a need for workshop-style training sessions to up-skill staff in the use of a validated nutrition screening instrument, accompanied with identification of clear nutritional management pathways and provision of appropriate nutrition resources. This approach has been shown by others to improve the success of implementation of a new process in the context of correcting malnutrition [31].

This study aimed to demonstrate feasibility of inclusion of a validated nutrition screening tool and accompanying nutrition resource kit for use in older patients attending general practice. Secondary aims were to assess whether a short training workshop improves nutrition-related knowledge of practice staff and to identify the extent of malnutrition in the patient group.

## Methods

Practices from the Illawarra and Southern Practice Research network were invited by email to participate in the study. Three practices responded, located in regional, rural and metropolitan areas respectively within the Illawarra and Shoalhaven Medicare Local catchment area of New South Wales, Australia. All general practitioners (GPs) (n = 19), general practice registrars (GPRs) (n = 2) and practice nurses (PNs) (n = 12) from the general practices were invited to participate in the study. Each general practice was allocated to use a different format of the MNA-SF to conduct nutritional screening based on feedback sessions in an original scoping study that identified preferences for formats that would best fit within the individual practice settings [30]: 1. Electronic format using an iPad; 2. Paper-based MNA-SF (to be completed by general practice staff); and 3. Self-administered version of the MNA (Self-MNA). Practice staff in the group allocated to the Self-MNA, were also required to complete the paper-based MNA-SF in order to determine whether there were discrepancies in the scoring. The MNA-SF categorises nutritional status into the following categories: well- nourished (score = 12-14), at risk of malnutrition (8–11), or malnourished (≤7).

A 60 minute training and discussion session on how to perform nutrition screening using the MNA-SF was provided by a dietitian (AHH) in group settings within each participating general practice. Participants completed a multiple choice questionnaire (MCQ) to assess their understanding of nutrition screening prior to participation in the training session, and again after three months. The MCQ was developed by the three nutrition content-matter experts in this study (AHH, KC and KW) based on key knowledge domains for assessing malnutrition in an older age group. Differences in scores were assessed statistically using a paired t-test. The IBM SPSS statistics software version 21 was used for statistical analysis. A Microsoft Power Point presentation, MNA-SF video [32] and case studies were incorporated into the training session. Each participant was also provided with a resource kit specifically developed for use in their own general practice. The kit included: a flexible tape measure (Seca 201, Hamburg, Germany); an information booklet (‘Managing malnutrition on our doorstep: A practical guide for general practice’ booklet) [33]; an MNA-SF tear off pad; a laminated chart of the recommended nutrition intervention pathways following nutritional screening; and copies of resources for distribution to patients. A portable stadiometer (Seca 217, Hamburg, Germany) and a digital flat scale (Seca 876, Hamburg, Germany) were provided to each practice.

The training session was conducted at each of the practices with consenting staff. The resource kit for patients included a non-perishable high energy and high protein foods leaflet, ‘Eating Well’ booklet [34], a relevant local council directory of nutrition-related services and available support services for older persons in the catchment area.

For a period of three months following completion of the training session, staff were encouraged to invite patients aged 75 years and older who were undergoing the 75 + HA and those attending the practice for consultations to additionally include the MNA-SF. Patients who agreed to be screened completed a written consent form. Patients who were identified to be ‘at risk of malnutrition’ or ‘malnourished’ were provided with a resource kit and other interventions, as outlined in the MNA-SF nutrition intervention pathway guide [35]. Additionally, it was advised that patients identified to be malnourished were referred to an accredited practising dietitian.

After three months, all participants (GPs and nurses) who attended the training session were invited to complete an open response questionnaire and in-depth individual interviews at each general practice. Participants’ perceptions about feasibility of administering the MNA-SF and the usefulness of the resource kit were assessed. The open-response questionnaire was developed and adapted by research dietitians (AHH, KC and KW) based on an Irish study in a community setting [31] which investigated the feasibility of implementing use of a validated nutrition screening tool, together with nutrition resources. The open ended questionnaire served as triangulation [36] for the in-depth interviews to further validate the results. The initial lines of inquiry, before individual exploration of interviewees’ responses, are shown in Table 1. All interviews were audio recorded, transcribed verbatim, coded into topics and thematically analyzed using qualitative analysis software QSR Nvivo version 10.

**Table 1:** **Initial lines of inquiry used in the in-depth interviews**

1.	Do you feel that the MNA-SF is better able to identify nutrition-related problems than using the 75+ Health Assessment alone?
2.	How do you feel that the MNA-SF was received by patients? Were they comfortable with answering the questions? And having measurements taken? (For general practice staff who completed MNA-SF)
	How was the MNA-SF received by patients who completed it themselves? (For patients from general practice who self-completed MNA-SF)
3.	Do you feel more confident to identifying malnutrition in older people since you attended the discussion session?
4.	Do you feel more confident in managing malnutrition in older people since you attended the discussion session?
5.	What additional resources or information should be made available about nutrition to staff working in General Practice?
6.	Is there anything else you would like to comment on regarding your experiences either in the discussion session or administering the nutrition screening tool?
7.	Has the nutrition screening increased your awareness about the prevalence of malnutrition and nutritional risk in general practice?
8	Do you think that the MNA-SF should be included as a formal part of the 75 + HA?

An iterative process for topics coding involved constant comparison, whilst themes emerged according to topics coding [37]. AHH undertook the initial topics coding through constant comparison, and AHH, KC and KW performed secondary coding and thematic analysis before circulation to all team members for further discussion and consensus. Quotes selected for reporting were chosen based on the most profound quotes in findings [38] and from various participants to demonstrate reliability of the analysis [39].

Ethics approval was obtained from University of Wollongong Health and Medical Human Research Ethics Committee (HE12/381).

## Results

Twenty-two general practice staff (n = 9 (47.4%) GPs, n = 2 (100%) general practice registrars and n = 11 (91.6%) practice nurses) from three practices participated in this study. Twenty-two post-intervention interviews were conducted with staff and 21 completed the open ended questionnaire. Four key themes were identified from the in-depth interviews regarding feasibility of administering MNA-SF. These themes were triangulated against the findings from the open-response questionnaire to search for disconfirming cases and refine the themes where appropriate. Most participants identified the MNA-SF as being either very helpful (n = 10) or helpful (n = 10) in identifying nutritional risk but one participant was unsure.

### Feasibility of performing MNA-SF

#### Theme 1: Ease of use

The MNA-SF scoring system was viewed by participants as being able to easily categorise a patient’s nutritional status. The nutrition intervention pathway also helped practitioners make decisions about how to further manage patients’ nutritional status.

*‘With the MNA (short form), you’ve got more of a tool, a guideline – you’ve got the numbers – so if they fit into that bracket, you know that there isn’t an issue. You’ve got a definite guideline’ (*PN2).

*‘It gave us a guideline for asking appropriate questions to identify those at risk of malnutrition whereas before we perhaps we wouldn’t have known which questions to ask and how to classify the significance of their responses’* (GPR1).

Feasibility of performing MNA-SF was further described in an open ended response:

*‘Useful screening tool-quick and easy to use’* (PN4).

*‘Quite efficient and provides a useful clinical indicator of nutritional risk’* (GP9).

#### Theme 2: Incorporation into existing practice

Practitioners provided overwhelming support for the incorporation of the MNA-SF into general practice software especially within 75+ HA templates.

*‘It would be better if it was integrated into our health assessment software’* (PN3).

*’It just has standardised it better for me and my practice – the way we do things. It’s more specific to nutritional risk than what’s in the existing (75+ HA) template. There’s more guidance for the person undertaking the assessment’* (GP4).

The MNA-SF was also described as being non-confrontational and similar to questions patients were being asked in the 75 + HA.

*‘I think that most patients would have been completely unaware that they were being receiving extra screening because a lot of it involves questions we would ask anyway’* (GP1).

Open-ended questionnaire responses also supported the interview result:

*‘It would be better to use the MNA-SF within Best Practice Software with automatic recording of the score (but this is not currently available)’* (GP1).

Practice nurses perceived MNA-SF as an ideal tool which can be fitted into a consultation:

*‘Easy to implement, can fit into a consultation time wise’* (PN5).

*‘It is short and simple and doesn’t take a great length of time, so it doesn’t confuse or exhaust the patient with questions, also it is easy to fit it into a visit’* (PN7).

#### Theme 3: Benefit to patients’ health

The MNA-SF can also serve as a nutritional awareness strategy for older adults as it provided an opportunity for staff to broach the topic of food and dietary intake.

*‘Some patients you’ll never think that they’ve got a malnutrition problem and after doing the screening you find that they are. It doesn’t mean that the patient is over-weight – he’s well-nourished’* (GP7).

This was further described in an open ended response:

*‘To keep elderly patients in optimal nutritional state as it so beneficial to health and recovery. Stop elderly people “slipping through the cracks” with malnutrition and not being noticed’ (PN11).*

#### Theme 4: Patients’ perception of MNA-SF

MNA-SF was well-received by patients as it involved simple questions.

*‘I think they were fairly happy to participate and they were usually quite thankful for the holistic care that we’re able to give. They received it quite well’* (GPR1).

Some staff expressed concern about patients being worried about the screening process and the purpose of screening, but reported that they were able to alleviate anxiety by further assurance and explanation regarding the purpose of the screening.

*‘I didn’t have any that refused it once I explained it. Some, at first, were a little bit hesitant but once we explained just what it was about they didn’t refuse it’* (PN7).

*‘I think there were a few times where they seemed relieved that it was only a short form I think, especially in a medical setting, often when you ask people to fill in a form they expect that it’s going to be long’* (PN8).

Practitioners also highlighted that obtaining written consent as part of ethics requirement brought more concern to patients.

*‘Written consent in order to ask questions of patients seemed quite onerous and I think the consent itself caused more distress to patients than the actual questions’* (GP1).

### Feasibility of using the resource kit

Two key themes emerged regarding the usefulness of the nutrition resource kit; applicability and improvement. The resource kit was found to be ‘very useful’ (n = 3), ‘useful’ (n = 11) and ‘not sure’ (n =  4) according to responses provided in the open-response questionnaire. Three participants didn’t provide any responses.

The resource kit was perceived as applicable for use in general practice.

*‘I think with that resource kit I think we’ve got pretty much all that would be relevant and useful and also applicable. I think any more than that would start to complicate the process too much for this setting’* (GP4).

*‘I think it’s a great kit for not just nutritionally at risk people; I think it’s a great kit that so many people over 75 would find really helpful. Not all – some people are functioning really well and don’t need any of that assistance but yes, I thought it had some really good information in it’* (PN8).

The main suggestion about how to further improve the kit related to availability of a shortened version provided in an electronic format that can be printed out for patients, as required, and annually updated to remain current.

*“Simple – just a sheet or a downloadable thing would be better that’s on our computer that we can just print off and hand to them”* (PN2).

Responses about electronic format of the resource kit were also supported in open response questionnaires.

*‘Electronic copies are more useful than paper copies’* (GP1).

### Nutrition screening knowledge scores

A statistically significant improvement in nutritional screening knowledge of general practice staff was found at the end of the three month test screening period, following training on given at baseline (p < 0.01) Mean score (standard deviation) improved from 5.7 (1.5) to 7.3 (1.1), whilst total score percentage increased from 51.8% to 66.4%.

Questions poorly answered before training related to the percentage of loss of body weight that would characterize risk of malnutrition, ideal BMI for older adults, and which oral nutrition supplements would provide an additional 400 kcal.

### MNA-SF scores

A total of 143 older adults were screened for nutritional risk using the MNA-SF across the three participating general practices. Table 2 details the nutrition screening results, according to allocation of the format of MNA-SF used and practice locations. Six (4.2%) and thirty-eight (26.6%) patients were identified as ‘malnourished’ and ‘at risk of malnutrition’, respectively and 99 (69.2%) were considered to be well-nourished. Discrepancies exist between scores obtained using the self-completed and practitioner-administered versions of the MNA-SF in group 3 as five patients (12%) were misclassified. Three patients had rated themselves as at risk, but staff had rated them as being well-nourished. One patient score was in the malnourished category but staff had scored them as well-nourished. Another patient that self-rated as well-nourished had been classified by staff as being at risk. Differences in scoring were identified in weight loss and body mass index questions.

**Table 2:** **Results of nutrition screening using different formats of the MNA-SF within general practices**

**Clinic**	**Version of MNA-SF**	**Malnourished**	**At risk**	**Well-nourished**	**Total**
1 (regional)	Electronic (iPad)	4	17	53	74
		5.4%	23.0%	71.6%	
2 (metro)	Paper based	2	12	13	27
		7.4%	44.4%	48.1%	
3 (rural)	Self-completed by patients	1	10	31	42
		2.4%	23.8%	73.8%	
	Paper based by staff	0	9	33	42
		0%	21.4%	78.6%	
All practices (Completed by staff)		6	38	99	143
		4.2%	26.6%	69.2%	

## Discussion

We are not aware of any previous reports of studies demonstrating the feasibility of routinely using a malnutrition screening instrument (MNA-SF) among general practice staff, using a mixed methods assessment approach. General practitioners and practice nurses identified the MNA-SF as an easy-to-use, systematic and quick tool that can categorise older patients according to their nutritional risk, which is consistent with findings from other countries [24,40]. The use of a validated instrument was valued as an objective way to facilitate nutritional risk identification in an open and non-threatening way. The intervention was associated with an improvement in practice capacity to identify malnutrition, indicated by an increase in knowledge scores after training and the three month trial. Practice staff identified that the six items in the MNA-SF were non-invasive and well-received by patient, which is an important criterion for a nutrition screening tool [41]. Our previous research has found that older patients may not be willing to divulge information to health care professionals about their dietary behaviours or social risk factors that impact on nutritional status due to fear of potential negative consequences, such as institutionalization and loss of independence [30].

General practice staff appreciated that different formats of the MNA-SF exist, either iPad- or paper based, and versions that can be self-completed by older adults or their carer. However, they emphasized that it is necessary to incorporate the MNA-SF into existing clinical software in order to integrate it into a patient’s electronic medical record. This would facilitate tracking of patients’ nutritional risk score over time, along with any changes in body weight. Further, electronic entry would enable direct linking of the MNA-SF result with the recommended nutritional management pathway, thus facilitating a clear decision-making process. In addition, the MNA-SF which has high sensitivity and specificity [22], is able to identify those individuals who might not be considered as at risk based on physical appearance, such as those who are obese.

While the self-completed version of the MNA-SF may be time-saving for practice staff, further investigation of its feasibility is warranted [42]. Our study indicates that incorrect estimation of body weight would bias the overall weighted scoring system and result in an incorrect nutritional risk assessment, as evident by score discrepancies between staff and several patients. However, the newly introduced Self-MNA has been demonstrated as having an acceptable inter-rater reliability when used by community-dwelling older adults [43]. It was our experience that practice nurses play a lead role in conducting routine nutritional screening in the general practice setting [20] and this is the group to be targeted for timely identification of malnutrition [24]. Higher participation rates from practice nurses than GPs likely reflect their commitment and/or capacity to undertake preventive care.

Findings from our study highlighted the benefit of nutrition education and training for general practice staff through demonstrated improvement in nutritional screening skills, knowledge and practice. In Ireland, an education programme incorporating guidance on using a validated nutrition screening tool was effective in up-skilling general practitioners and practice nurses [31]. That programme required dietitians to engage with general practice staff in a one hour session and to assess their knowledge, which was consistent with our approach. Another study in UK identified that nutrition training led by dietitians improved practice nurses’ nutrition knowledge which also contributed to being more confident in providing simple nutrition advice to patients [44]. A systematic review has further identified positive dietary changes in older adults who have received nutritional advice from health care professionals [45].

The nutrition resources for patients were found to be useful to deliver nutrition messages. General practice staff felt that they did not possess the necessary skills to effectively manage malnutrition and therefore felt more comfortable to hand out the nutrition resources to patients. This finding is similar to that reported more than ten years ago among Australian general practitioners [46] who preferred to hand out nutritional resources to patients rather than playing a key role in their nutritional management. A need to refer malnourished patients to a dietitian for further assessment and management was acknowledged. Our study did not attempt to transfer these specialised skills to general practice staff, but rather aimed to encourage greater awareness of nutrition-related issues and the need for opportunistic routine nutrition screening in all older patients.

Our findings demonstrate that malnutrition remains a problem in community-dwelling older adults attending general practice, as reported by others [47]. An Australian study of home nursing service clients showed higher rates of malnutrition with 8% of older adults being malnourished and an additional 35% being categorised as ‘at risk of malnutrition’ [8], while another study of older people receiving home care services in South Australia reported a higher prevalence of nutritional risk [9]. Pooled global estimates using the full MNA assessment [23] rather than the MNA short form screen, in community dwelling older adults, indicate that 32% of this group are ‘at risk of malnutrition’ and 6% are malnourished [48]. In our study, less than 10% patients from the same age group in each general practice participated in the screening and 27% of them were identified as at risk. Both malnourished and ‘at risk’ community-dwelling older adults have a higher risk of hospital admissions, with longer hospital stays than their well-nourished counterparts [9] which indicates that targeting the ‘at risk’ group to prevent further health deterioration is warranted [15].

Preventive health is a major focus of primary health care reform in Australia, especially in older adults [49]. General practice is recognised as an appropriate setting in undertaking timely identification of malnutrition through nutrition screening [50]. The MBS-funded health assessment for older adults aged 75 years and older (75 + HA) is an initiative aimed at improving the identification and management of medical and non-medical problems [12]. We provide evidence that nutrition screening in older adults can become routine practice if it is incorporated within this annual health assessment [30,51], a concept that is also advocated by international authors [18,41]. An additional opportunity is the recent (July 2012) introduction of the electronic patient health record by the Australian Department of Health [52]. Incorporating patients’ nutritional screening scores into electronic medical records would improve interdisciplinary care between general practitioners, practice nurses, dietitians and other allied health professionals, as well as allow for better communication between sectors of the health care system. Older adults are susceptible to rapid nutritional decline [53], especially following hospital discharge after an acute illness when they may be referred home with little nutritional support, resulting in hospital readmissions, poor quality of life and mortality [4,5].

Additionally, there may be financial incentives for General Practices to incorporate nutrition screening within the 75+ Health Assessments as a means of improving holistic care for older patients within nurse-led consultations that attract Medicare rebates. Older patients contribute significantly to non-billable time for GPs. A continuous national study of general practice activity in Australia reported that 12% of patients had non-billable time spent between previous and current visits over the period of a year [1]. This can be extrapolated to a national figure of 16.3 million encounters annually, representing 2.7 million hours of GP’s time or an average of 2.5 hours per week, equivalent to 8.6 standard consultations per week. This translates to a substantial loss of potential income, of approximately $15,000 per General Practice per year. Importantly, the likelihood of non-billable patient encounters increased dramatically with patient age and with the management of at least one chronic health condition.

Limitations to this study include a small number of health care practitioners recruited from only three general practices in a single health district using a convenience sampling technique, and a relatively short duration of the intervention period. The questionnaire that was used to assess change in knowledge of practice staff following upskilling and training was not trialled before use, which may limit its content validity, however overall score improved and it was the relative change that was important to demonstrate. The requirement by ethics to obtain written consent from patients for nutrition screening may have reduced their participation rate rather than if screening had been offered as a usual part of the model of care, however this cannot be confirmed. A strength of the research was inclusion of three practice locations from a metropolitan, regional and rural area, as well as the mixed methods approach that included qualitative as well as quantitative data. This study represents the second of a three-phase participatory, action-based research project that has been designed to improve malnutrition identification in older adults through nutrition screening and appropriate nutrition intervention in the Australian general practice setting. The first exploratory phase [30] informed the model tested here, while the final phase will explore the practicality of incorporating an electronic format of the MNA-SF into clinical practice software, as well as investigate whether nutritional screening impacts on patient outcomes.

## Conclusion

Implementing routine nutritional screening in general practice is feasible through the use of an easy, systematic tool, the MNA-SF provided it is accompanied with training and provision of relevant patient resources for use by general practice staff. Improvement in nutritional screening skills and knowledge can be achieved by up-skilling general practice staff with practical guidance. Timely nutrition intervention for the ‘malnourished’ and ‘at risk’ group could prevent further deterioration of nutritional status. Future incorporation of the MNA-SF within general practice clinical software was viewed as the most feasible format as the screening score could be linked with patients’ medical record and incorporated into annual health assessment.
